# Relationships between Parental Socialization Styles, Empathy and Connectedness with Nature: Their Implications in Environmentalism

**DOI:** 10.3390/ijerph16142461

**Published:** 2019-07-11

**Authors:** Daniel Musitu-Ferrer, Celeste León-Moreno, Juan Evaristo Callejas-Jerónimo, Macarena Esteban-Ibáñez, Gonzalo Musitu-Ochoa

**Affiliations:** Department of Education and Social Psychology, Pablo de Olavide University, 41013 Seville, Spain

**Keywords:** parental socialization, adolescence, empathy with nature, connectedness with nature

## Abstract

Parents exert a strong influence on several adjustment outcomes. However, little is known about their influence on adolescents’ connectedness with the environment. This study examined the relationships between parenting styles, empathy and connectedness with the environment. The two-dimensional socialization model was used with four resulting styles: Indulgent, authoritative, neglectful and authoritarian. The sample comprised 797 adolescents (52.7% girls) from six public secondary schools who were aged between 12 and 16 years (*M* = 13.94, *SD* = 1.28). The results showed significant relationships between parental socialization styles, empathy and connectedness with nature. It was also observed that adolescents from indulgent and authoritative families showed higher levels of empathy and connectedness with the environment than adolescents raised by authoritarian and neglectful parents, with males from such families consistently presenting the lowest levels of empathy and connectedness, which was not the case among women. Additionally, women, regardless of the parental style in which they had been educated, showed greater cognitive and emotional empathy with the natural environment, while adolescents raised in indulgent and authoritative families displayed higher levels of empathy and connectedness than those with authoritarian and neglectful parents. These results suggest that indulgent and authoritative styles are stronger enablers of empathy and connectedness with nature.

## 1. Introduction

Today’s serious environmental problems, such as the pollution of the seas, the depletion of the ozone layer, global warming, the destruction of forests, climate change, and the scarcity of energy and water are limiting the ability of human and non-human living beings inhabiting this planet to survive and also constitute one of the greatest challenges facing the world’s population today [1,2]. This dramatic situation has been affecting citizens, albeit very slowly, in the form of greater commitment and respect for the environment and more pro-environmental and sustainable behavioural changes [3,4,5]. In this sense, much research has been carried out in recent decades with the aim of answering the environmental problems that affect us and promoting a change of mentality regarding the relations between human beings and nature—having a regard to such environmental education is crucial in order to transmit the principles of sustainable development [6,7,8,9,10,11].

The main aim of environmental education is to educate citizens committed to preserving and respecting the natural environment so that they can understand and internalize their relationship and reciprocal dependence [12]. However, when analysing the proposals that are being formulated to address environmental problems, it has been observed that these are mainly technological [13], and very few are based on education, which is surprising and alarming because there is an almost unanimous consensus among researchers and many citizens that education is indispensable for sustainable development [14,15]. Moreover, environmental problems are increasingly not perceived as a technological problem but also, fundamentally, as a problem of behaviour and perception [16]. Therefore, solutions to environmental problems are not found so much in technology as in the modification or change of human behaviour [17,18], with an emphasis on psychosocial and educational processes. For this reason, understanding the processes and factors constituting pro-environmental behaviour, together with their most significant predictors, is important to achieve a more sustainable future [19]. This study analyses the socialization processes of parents, in which family, empathy, and connectedness with the natural environment are important protagonists in the educational process and, consequently, in the protection and care of the environment. These protagonists are the most important factors/predictors, along with pro-environmental behaviours and attitudes [20,21,22]. This research aimed to answer the following question: What are the relationships between parental socialization styles and empathy and connection to the natural environment?

### 1.1. Parental Socialization Styles

Research on family socialization has mainly focused on analysing the practices or strategies used by parents to achieve the internalization of beliefs, values and normative behaviours in a given society, as well as the impact that different forms of parental socialization have on children, i.e., on their personal, prosocial and social adjustment [23,24,25].

The operational definition, i.e., the relationship between parental socialization styles and the adjustment and quality of life of children, has been analysed following a two-dimensional model [26], in which the dimensions of “responsiveness/demandingness,” also called “acceptance/implication” and “severity/imposition” [27,28], were assumed to be orthogonal [29] and to reflect two behavioural models with respect to socialization [24]. The “acceptance/implication” dimension refers to understanding, affection, warmth and support for the autonomy of children, and the “severity/imposition” dimension refers to assertiveness and the strict definition of limits. Based on these two orthogonal dimensions [27,28] four parental styles are obtained: Authoritative—high acceptance/implication and high severity/imposition; indulgent—high acceptance/implication and low severity/imposition; authoritarian—high severity/imposition and low acceptance/implication; and neglectful—low acceptance/implication and low severity/imposition [26,27,28,30,31,32]. One of the main questions that researchers have been asking in recent years regarding parental socialization styles and their effects is the following: What is the optimal socialization style for children’s adjustment? There is no unanimous answer to this question.

In scientific literature regarding parent–child relationships, one of the most consistent results since Baumrind’s first studies [33,34] with middle-class American families is that children raised in authoritative families present better psychosocial adjustment than those raised in indulgent, authoritarian and negligent families [26,35,36,37,38], and it is in these latter families that children present the lowest levels of adjustment [28]. However, these results are not replicated in all cultures, and there are frequent inconsistencies [39] in the sense that the authoritative style is not always related to the better psychosocial adjustment of children. For example, studies conducted with Asian minorities in the United States have observed that the use of an authoritarian style is associated with better academic outcomes—which are a measure of adjustment—than with other styles [40,41,42,43]. The authoritarian style has also been positively related to the mental health of adolescents in Arab societies [44]. Likewise, and with respect to other cultural contexts such as Spain, indulgent styles have been related to better or similar adjustment of adolescents when compared with authoritative styles [32,45]. Similar results have also been obtained in Italy [46,47] and Brazil [48,49]. In Germany, Barber, Chadwick, and Oerter (1992) [50] found that, in German society, greater academic self-esteem was more strongly related with expressions of affection and parental support than with the authoritative style. An interesting study by Wolfradt, Hempel, and Miles (2003) [51] reported that adolescents who perceived their parents as permissive (indulgent) showed better psychosocial adjustment, less depersonalization and lower anxiety, while higher scores were observed in families that employed authoritarian styles. Mean scores were obtained in families with authoritative and negligent styles. However, the results presented in other studies differ from those described previously insofar as when adolescents come from indulgent families they are less involved in school, show greater failure and also show more behavioural problems [28,52]. These inter- and even intra-cultural differences seem to confirm the hypotheses formulated by various authors—namely that the same parenting practices may be associated with different socialization objectives [53], and that the meaning of socialization practices may be different according to culture [39,54,55,56].

### 1.2. Family Process and Natural Environment

The factors that influence different expressions and behaviours related to the environment can broadly be grouped into the following categories: Individual, family, educational and situational factors [57,58]. In the field of Education and Environmental Psychology, all these areas have been widely studied in relation to pro-environmentalism, with the exception of the family sphere and its functioning and processes. Through socialization processes, parents exert a significant influence on values, attitudes and behaviours towards the natural environment [59,60]. The internalisation of pro-social values, such as self-transcendence (benevolence and universalism) and conservation (conformity, security and tradition), are associated with indulgent and authoritative socialization styles [49,61,62]. These values are, in turn, those most related with pro-environmentalism [63,64], defined as any behaviour that benefits nature and improves environmental quality [65,66]. Parents (implicitly or explicitly) attempt to pass on their values to their children, which implies a positive correlation between the value priorities of children and their parents, including nature-related values [18,67,68,69,70,71].

One theory that may facilitate the understanding of these processes that take place between parents and children would be the empowerment theory. In this theory, a distinction is made between empowerment processes and the results deriving from these processes. One such process would be parental socialization, including actions, activities or structures that allow us to set in motion efforts to obtain control and resources that satisfy individual and social needs. The results of empowerment refer to the operability of this process in such a way that the effects/results produced by these processes can be studied [72,73,74]. In the stages of child and youth development, empowerment at the individual level (micro level) refers, from an ecological standpoint, to personal interactions and relationships in the most microsocial sphere of the individual. In this sense, the influences of family and school are fundamental in the enhancement of resources such as empathy and connectivity, as well as in the confidence with which the individual faces the daily challenges of life with a perception of self-efficacy and a certain level of self-esteem [75,76,77].

Recent research has highlighted these theoretical relationships between family functioning, attitudes towards the environment and concern and sensitivity for environmental problems [59,78,79,80], which are normally explained through two potential routes: Modelling and direct socialization styles (e.g., recycling, controlling water and energy consumption and showing respect for the environment). Through these processes, parents influence aspects related to their children’s environment, such as respect and pro-environmental behaviours [59,60]. They are also strongly influenced by norms prevailing in the family and the way in which they manifest themselves in their parents’ behaviour [59]. In one interesting study, Grønhøj and Thøgersen [67] found that parental styles that enhance autonomy also contribute to the development of intrinsic motivation to participate in pro-environmental behaviours. This is an interesting result insofar as parents’ support of autonomy is related to so-called functional socialization styles such as the indulgent style and the authoritative style [31,32,56]. In addition, Erhabor and Oviahon [81] observed that when family functioning is positive, children show respectful and caring behaviour towards the natural environment. Karmakar [82] found that when parental styles are consistent with pro-social behaviour, children are more pro-socially motivated, and this behaviour is known to be related to pro-environmental conduct [14,83,84]. These studies highlight the importance of family in pro-environmental attitudes and behaviours. However, very few studies have analysed the relationships of parental socialization styles with the environment, particularly with empathy and connectedness to the natural environment, although much has been explored in the field of interpersonal relationships [56].

### 1.3. Empathy and Connectedness with Nature

Empathy to nature is a continuity of interpersonal empathy, although it is a different construct and can never be reduced exclusively to said form of empathy [22]. Similar terms are used, such as “empathic concern” [85], “sympathy” [86,87], “adoption of perspective” [88] or “compassion” [89], all with the same common denominator, namely to share emotions with others or with nature [90,91,92,93]. Empathy with the environment is defined in the context of interpersonal relationships as an emotional reaction congruent with the emotional state of the other and identical or very similar to what the other person is feeling or could feel [86,91,94]. There is a certain consensus to consider empathy as the understanding and exchange of the emotional experience of another person or object [92]. Its importance in the field of education and environmental psychology was highlighted masterfully by Hoffman [95] in the following terms: It would have been impossible for human beings to have survived as a species if we had all been concerned exclusively with ourselves (p.1). However, empathy is not only the interpersonal ability to put to one side the egocentric impulses of inter-subjective identification, it is also the ability to identify with the environment, with the evolution of the biosphere understood as the shared place of all species, whose fate we share [96].

Studies on empathy have generally followed a two-dimensional perspective: The cognitive perspective—mental perspective taking—[88,97,98], and the emotional perspective—the vicarious sharing of emotion—[87,99,100]. Recent studies have found that “both cognition and affect are important, but more research is needed to determine how they work together” [101]. Some authors also consider it to be a trait—dispositional empathy—or state—situational empathy—in both interpersonal relationships and relationships with nature [22]. An essential aspect of empathy is that it can be strengthened during our evolution and through our interactions [21]. Therefore, in this context, empathy towards the non-human world would mean acknowledging the needs of animals and nature in general, the importance of their survival, and showing interest in their well-being [21,102].

Connectedness is a variable that, like empathy, has a great capacity for predicting pro-environmental behaviour. As a result, it has begun to play a significant role in environmentalism in recent years [20,22,103]. It is defined as the degree to which human beings integrate nature in their cognitive representation of self [104]. Mayer and Frantz [105] later complemented this definition by incorporating affective and experiential connectedness with nature and defined it as human beings’ emotional connectedness with the natural world. Extending the self-concept to include nature creates a deep sense of proximity and belonging [106] and, consequently, of connectedness with all living beings [107], to the extent that nature and self are perceived as one and the same [108,109]. Once nature has been incorporated in self, it is easy to understand that any mistreatment of nature is like oneself [105,109,110,111,112]. Individuals with this connectedness perceive themselves as part of a larger natural community with which they feel great affinity. They feel that they belong to the natural world as much as that world belongs to them. They are also convinced that their own well-being is linked to the well-being of the natural world [20,105].

In relation to gender, much research has concluded that women show greater connectedness and empathy with the natural environment [22,113,114,115], a greater commitment to environmental protection [116,117], and more pro-environmental behaviours such as recycling and energy saving [118] than men. These results coincide with those reported in research at the end of the last century, one example being the interesting meta-analysis of the period between 1988 and 1999 carried out by Zelezny, Chua, and Aldrich (2000) [119] in twelve countries and which concluded that a gender difference exists in terms of pro-environmental attitudes and behaviour between groups and between countries, with higher scores for women. Psychosocial theories of gender socialization and gender role have been used to explain these gender differences in environmentalism [114,120] concluding that women’s socialization is more oriented towards aid and cooperation and more pro-social and empathic, in turn implying greater empathic concern for other animals and nature in general [92,120,121]. In addition, and with respect to empathy with the natural environment, gender differences between men and women have been explained according to the theory of social dominance orientation (SDO) [122], which considers that women are more concerned and committed to the natural environment than men because they also have a lower SDO and greater empathy [118,123]. However, the results regarding gender in environmentalism are far from conclusive, as reported by Tam (2013) [22]—gender studies in environmentalism are still in their initial stages, and much remains to be done.

### 1.4. The Present Study

This study aimed to analyse the relationships between parental socialization styles (authoritarian, neglectful, authoritative and indulgent), empathy, and connectedness with nature among Spanish adolescents. Parents are identified as the main socializing agents even during adolescence [124]. Significantly, despite adolescent deviance against adult standards and social values (e.g., environmental values) being related with negative peer influence, family may either constitute a risk of or serve as protection against adolescent vulnerability, usually identified in early and middle adolescence [124,125,126]. Moreover, it has been widely shown in the field of psychology and education that parental socialization styles are related to interpersonal empathy [127,128,129], altruism [130,131], and pro-social behaviour [132,133,134,135]. These variables are also related to pro-environmental behaviour [14,83,136], pro-social values [61], family connectedness [137] and with connectedness and autonomy [138,139]. However, there is a significant gap in this field of study, and more in-depth knowledge in this area would be very productive for psychology and environmental education [59,60,67,81]. The semantic proximity of these concepts with empathy and connectedness with the environment prompts our consideration of the first hypothesis: 

H1. Styles of parental socialization will be significantly related to empathy and connectedness with nature.

In terms of the results of parental socialization, there is broad consensus among researchers regarding two ideas: 1) Cultural differences determine the protagonism and effectiveness of different parental styles [31,32,140], and 2) indulgent and authoritative styles generally have the strongest influence on the resources and well-being of adolescents. The authors believe it is plausible for these same effects to take place in relationships of empathy and connectedness with the environment, prompting us to form the second hypothesis: 

H2. The indulgent and authoritative styles are the ones most related to empathy and connectedness with nature.

Regarding gender, it has been observed that empathy, connectedness, attitudes and care for the environment are stronger in women who, in turn, tend to show higher levels of empathy and connectedness with nature [113,115,141], a more positive attitude towards the environment, and a greater involvement in pro-environmental behaviours [22,118,142]. Nevertheless, little research has been performed in this field. The authors of this paper believe that exploring these relationships will enrich current knowledge of these relationships. Hence, the following hypothesis is proposed:

H3. Indulgent and authoritative socialization styles will be associated with greater empathy and connectedness with nature in women, as compared to men.

## 2. Materials and Methods

### 2.1. Participants

The size of the sample was determined based on a prior analysis of the power assuming a mean effect size (*f* = 0.14). The software program G*Power was used [143,144]. Type-I and Type-II errors were set within the usual limits—*α* = 0.05 and *β* = 0.90—for the univariate *F*-tests among the four parental styles. The results of a prior power analysis indicated a minimum sample size should be 728 participants [144,145]. A total of 797 adolescents (52.7% girls) participated in this study. They were enrolled at 6 public high schools in the province of Alicante (Spain). The participants were aged between 12 and 16 (*M* = 13.94, *SD* = 1.28). The post hoc power of any *F*-test for the four parenting styles (*f* = 0.140; α = 0.05) was 0.93 (β= 0.07) [143,146,147]. A power sensitivity analysis indicated that the minimum effect size minimum that could be detected was 0.139.

### 2.2. Procedure

In terms of the procedure, once the six centres had been selected, contact was made with the staff. With the staff’s help, an informative seminar was organized for teachers and parents to explain the aims of the project, accept operational suggestions, and answer any questions. Permission was also requested from the parents of the students, as well as the collaboration of the teachers. The questionnaires were administered in the usual classrooms during a regular class period. All the adolescents were informed that participation was voluntary and confidential. The study complied with the ethical values stipulated in the Declaration of Helsinki [148].

### 2.3. Materials

Parental socialization: The Parental Socialization Scale (ESPA29) [31] evaluates parental socialization styles in different scenarios representative of everyday family life. The performance of fathers and mothers was assessed separately in 29 significant situations through 232 items: 16 alluded to adolescents’ behaviours referring to family norms (e.g., “If I order and take care of things in my house”) and 13 referred to behaviours contrary to these rules (e.g., “If I tell a lie and they find out”). From these situations, a global measure was obtained for each parent in the acceptance/implication dimensions, α = 0.90; and severity/imposition dimensions, α = 0.96. The socialization style—neglectful, authoritarian, indulgent and authoritative—was typified based on the scores obtained in the two dimensions [31,32]. The Cronbach’s alpha reliability coefficient scale was α = 0.95. In line with previous parenting studies, the socialization style—neglectful, authoritarian, indulgent and authoritative—was typified based on the scores obtained in the two dimensions (i.e., acceptance/involvement and strictness/imposition) by the median split [31,58,149]: Authoritative parenting (above the median for both acceptance/involvement and strictness/imposition), neglectful parenting (below the median for both parenting dimensions), authoritarian parenting (above the median for strictness/imposition, but below the median for acceptance/involvement), and indulgent parenting (above the median for acceptance/involvement but below the median for strictness/imposition). In line with previous parenting studies, adolescents rated parenting measures (i.e., acceptance/involvement and strictness/imposition), and these ratings for mother and father were averaged (see [27,150]).

Environmental empathy: The Environmental Empathy scale (EES) [141] consists of 11 items that measure the emotional and cognitive empathy of adolescents with nature, with responses ranging from 1 to 5 (never, rarely, sometimes, frequently, and always). The 11 items on this scale are grouped into two dimensions: Emotional empathy is measured with six items (e.g., “I feel good if I am in a natural environment that is protected and cared for”), α = 0.83. Cognitive empathy with nature is measured with five items (e.g., “When a friend performs an act that harms the environment, I try to understand his/her reasons”), α = 0.79. The Cronbach’s alpha reliability coefficient scale was α = 0.88.

Connectedness to nature: The Connectedness to Nature scale (CN8) [141] consists of 8 items that measure connectedness with nature with responses ranging from 1 to 5 (never, rarely, sometimes, frequently, and always) (e.g., “I am aware that some of my behaviours have a negative effect on the natural environment”). The Cronbach’s alpha reliability coefficient scale was α = 0.86.

### 2.4. Analysis Plan

First, the cross-distribution of parental styles according to gender was calculated. Then, a multivariate variance analysis (MANOVA) was performed using the Statistical Package for Social Sciences (SPSS) version 20 (Pablo de Olavide University, Seville, Andalusia, Spain). To analyse the relationships between parental socialization styles and empathy and connectedness with the natural environment, a multivariate factorial design (MANOVA 4 × 2) was applied, in which connectedness with the natural environment and the two dimensions of empathy with the natural environment were the dependent variables, and parental socialization styles (neglectful, authoritarian, indulgent and authoritative) and gender (male vs. female) were the independent variables. Likewise, subsequent ANOVAs and Bonferroni post-hoc tests were applied to analyse the differences between means. This study also considered the same traditional design and robust statistical analyses as other seminal studies (i.e., [58]). These statistical procedure was able to examine the impact of the four parenting style and all of their possible combinations on the factorial design with different adjustment criteria [27,32,45,151,152]).

## 3. Results

First, the distribution of parenting styles with gender and age groups was calculated (see Table 1). Table 1 shows the adolescents classified according to each parental socialization style (neglectful, authoritarian, indulgent and authoritative). 

Then, the MANOVA (multivariate analysis of variance) statistical technique was used with the SPSS statistical package (version 20). Multivariate factorial design (MANOVA, 4 × 2) was applied with the dimensions of parenting styles (neglectful, authoritarian, indulgent and authoritative) and gender (male and female) as independent variables to analyse potential interaction effects. Subsequently, univariate *F*-tests were carried out to study the differences between the dependent variables, and a Bonferroni post-hoc testing was performed. As shown in Table 2, the factorial MANOVA showed significant differences in the main effects of the parental socialization styles (Λ = 0.919, *F*(9, 1915.502) = 7.514, *p* < 0.001), and gender (Λ = 0.948, *F*(3, 787) = 14.290, *p* < 0.001). A significant interaction effect was also observed between parental socialization styles and gender (Λ = 0.987, *F*(9, 1915.502) = 1921, *p* < 0.05).

### 3.1. Parental Socialization Styles and Connectedness and Empathy with Nature

In terms of the parenting styles variable, the results showed significant differences in connectedness with nature (*F*(3, 793) = 11.53, *p* < 0.001), emotional empathy with nature (*F*(3, 793) = 16.37, *p* < 0.001), and cognitive empathy with nature (*F*(3, 793) = 12.99, *p* < 0.001). Compared to adolescents from neglectful and authoritarian families, adolescents from indulgent and authoritative families scored the highest scores in relation to connectedness, as well as in the two dimensions of empathy with nature(see Table 3). 

### 3.2. Sex and Empathy with Nature

As shown in Table 4, women obtained higher average scores than men in both dimensions of empathy with the environment: Emotional (*F*(1, 795) = 46.40, *p* < 0.001) and cognitive (*F*(1, 795) = 19.04, *p* < 0.001). No significant effects were observed for gender in connectedness with nature. 

### 3.3. Parental Socialization Styles and Sex and Emotional Empathy with the Natural Environment

**Figure 1:**
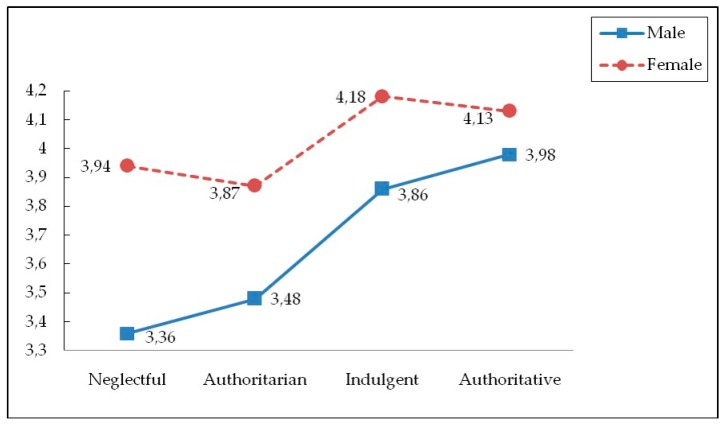
Interaction effect parenting styles x gender and emotional empathy to natural environment.

As Table 5 and Figure 1 presents, a statistically significant interaction effect was obtained in emotional empathy with nature (*F* (3, 789) = 2.760, *p* < 0.05, η^2^ = 0.010). It was observed that the highest scores in emotional empathy with the environment corresponded to men and women raised in authoritative and indulgent families, whereas the lowest scores were observed in those with authoritarian and neglectful parents. Women and men from authoritative families did not differ in terms of emotional empathy, but differences were observed between men and women with indulgent, neglectful, and authoritarian parents, with lower scores for men. In women, emotional empathy was independent of the socialization style, but this was not the same in men who presented significant differences in relation to empathy between those raised by indulgent and authoritative parents on the one hand and those with authoritarian and neglectful parents on the other.

## 4. Discussion

The aim of this study was to analyse the relationships between parental socialization styles and empathy and connectedness with nature in school-aged adolescents, taking into account gender. To achieve this objective, a MANOVA 4 × 2 was carried out. The first observation was that the parental socialization styles mainly affected connectedness and empathy—cognitive and emotional—with nature, thus confirming the first hypothesis in which it was stated that styles of parental socialization will be significantly related to empathy and connectedness with nature. This result is considered important because very few studies have explored the relationships between these dimensions and also because it makes a significant contribution to knowledge on family and nature—in this case, in the specific field of parental socialization processes. This result could be explained following the theory of empowerment, which suggests that in intervention programs in different scenarios transited by human beings (all mutually interdependent) these clearly differentiate strengthening processes from results, which are the effects deriving from the aforementioned processes [72,73,74]. In this sense, parental socialization processes are some of the most important and significant processes due to their effects on and implications for the health and adjustment of children and adolescents [26,31,32,153]. These results could suggest that parental socialization styles do not only influence children in the individual [47,154], family [155] and social spheres [156], but also in their relationships with nature, which would constitute an extension of the family system. In terms of the results of socialization, this would also represent an extension of individual life related to health and well-being. In fact, numerous studies have described the relationships between empathy and connectedness with nature environment and health [157], well-being [158,159] and happiness [160]. Recent research has already highlighted both the lack of research in this area and the interest in incorporating the family more fully because of its role in the pro-environmental behaviour of children and adolescents [18,81,82].

Secondly, it was observed that the indulgent and authoritative styles enhanced empathy and connectedness with nature most strongly, compared with the neglectful and authoritarian styles, which had the least impact, thus confirming the second hypothesis in which it was established that indulgent and authoritative styles are the ones most related to empathy and connectedness with nature. These results are in line with those reported in numerous studies in the field of psychological research that have highlighted the importance of these styles in the enhancement of resources in children, such as self-esteem [161], social and emotional competence [161,162], values, and, consequently, their adjustment [45,163,164,165]. The authors believe that these results are significant insofar as they illustrate that an important requirement in the transmission of values and attitudes—as well as in the enhancement of empathy and connectedness with the environment—is that the family functions [82]; these types of families normally uses functional socialization strategies, such as the indulgent and authoritative styles, which have been normally considered the most powerful. with certain variations depending on the culture [26,31,32,61]. These families generate a strong sense of attachment in children, and so-called “epistemic trust” is built within the family, enabling fluid, direct and unfiltered communication, as well asthe transmission of rules and information relevant for socialization [166,167]. However, it is important to bear in mind that the results obtained in this study may not be replicated in other cultures due fundamentally to the different meanings that different styles of socialization may have [39,54,55,56]. In addition, Rao, McHale, and Pearson (2003) [53] observed that inter- and even intra-cultural differences seem to indicate that the same child-rearing practices may be associated with different socialization objectives. Based on the results obtained in this and previous research, it may be concluded that parents transmit their pro-environmental attitudes and behaviours to their children through socialization and modelling [18,59,168,169] and through communication and interaction with them [67,170]. For example, recent studies have reported that family and friends play an important role in relation to concerns regarding climate change by facilitating communication between family and friends and helping in the search for information [78,79,80,171].

Finally, and as predicted in the third hypothesis that indulgent and authoritative socialization styles will be associated with greater empathy and connectedness with nature in women as compared to men, it was observed that the highest scores in emotional empathy with the environment were obtained in men and women from authoritative and indulgent families, compared with the lowest scores for those with authoritarian and neglectful parents. Women and men from authoritative families did not differ in terms of emotional empathy, but differences were observed between men and women with indulgent, neglectful, and authoritarian parents, all of which had lower scores for men. In women, emotional empathy was independent of the socialization style, but the same did not occur in men. The fact that women displayed greater empathy with the environment simply confirmed the results obtained in numerous studies that highlight women’s greater sensitivity, pro-environmental attitudes, and concern for the environment [119,172,173,174,175,176]. These differences between men and women in environmentalism have been explained through their gender roles and socialization, considering that women devote their leisure time to deeply socialized roles, strongly associated with the “ethic of care” [177] (p.147). The underlying idea is that this greater interpersonal empathic concern is acquired by women, and gender role experiences and expectations give rise to a stronger empathic concern regarding other animals and nature [123].

One interesting finding showed that emotional empathy in women is independent of each parental socialization style, in contrast to men, with lower scores for emotional empathy with the environment being obtained for men raised by authoritarian and neglectful parents. These differences between the genders suggest that one must look for other explanations to those mentioned previously that are not merely social, such as the biological aspect [171,178] or the fact that women have more future-oriented thinking, which could account for their greater concern and care for the environment [179]. Regardless of whether these or other explanations account for these differences, the fact is that gender differences in environmentalism are far from being resolved. 

In the authors’ opinion, this research has interesting practical implications. The fact of discovering that variables such as empathy and connectedness with the environment—which are important predictors of pro-environmental behaviour—are related and promoted through the most positive styles of parental socialization according to each culture can help to enrich intervention programmes both in the family and at school. These results suggest that positive parenting programmes include the idea that the effects of positive relationships between parents and children include connectedness and empathy with the environment and, obviously, greater involvement with caring for nature. Thus, the idea that positive parenthood has more beneficial implications than just achieving adequate psychosocial adjustment of children is being filtered out and reinforced in parents. Moreover, through such information, parents can show a greater motivation and willingness to talk to their children about the serious environmental problems that afflict us, such as responsible consumption, recycling, the pollution of the seas, and excessive energy and water consumption. In this sense, it has been observed that in properly functioning families, in addition to promoting positive socialization, communication is fluid and can help with multiple existing problems including those highlighted previously—namely, the deterioration of the environment. Therefore, good communication has positive effects on pro-environmentalism [59,67]. Attention should be drawn to the idea that interpersonal empathy and social connectedness have connotations very closely related to empathy and connectedness with the natural environment [20,22,180,181,182,183] and which are also related to parental socialization styles [47,61]. Lastly, school is an ideal place to transmit to parents the benefits of an empowering socialization, as well as inviting them to forge a stronger connection with nature by fostering walks through parks, valleys and mountains that, in addition to promoting connectedness and empathy with the environment, also have great benefits for the health and satisfaction with life of parents and children, as well as in parenting. Engaging in such simple activities is nothing more than responding to a call for help from nature, of which we are all part.

This study, like all empirical research, has its strengths and weaknesses. Its strengths include the following: 1) Its design, which incorporated the parental socialization styles often used in psychology but very little in environmentalism, together with very significant dimensions in Environmental Psychology, such as empathy and connectedness with nature. 2) The results obtained broaden knowledge in the field of Education and Environmental Psychology, in addition to promoting new theoretical debates on the role of family and gender in environmentalism. 3) The study presents interesting proposals to encourage empathy and connectedness with the environment through socialization styles and future lines of research incorporating family through classical socialization styles, empathy, and connectedness with nature. Its weaknesses include the following: 1) The selection of the sample was not probabilistic, which could influence the general application of these results to the population; 2) In addition, given the cross-sectional nature of this study, it cannot be excluded that the relationships between the examined variables may be bidirectional. 3) The results were obtained from a single source: Adolescents. Future studies should focus on obtaining information from more sources, such as peers and parents. However, these self-reported measures have been found to be more valid and reliable than measures obtained from parents [184,185]. Future research should also incorporate objective pro-environmental behavioural measures such as waste recycling, energy saving, and moderate consumption in order to obtain more in-depth knowledge of parental socialization and the care and protection of the natural environment, in addition to controlling social desirability and hypocrisy in self-reporting measures. However, despite these limitations, the authors believe that the results obtained in this study are important and significant insofar as parental socialization styles are incorporated together with empathy and connectedness in the research on the natural environment, and we hope these findings will benefit environmental education programs aimed at parents and school teachers. More in-depth research into these relationships may significantly enhance our understanding of how to involve adolescents more in pro-environmental behaviours. In the 1990s, Clayton [186] claimed that only the experience of connectedness will save the earth and us with it.

## 5. Conclusions

In the authors’ opinion, the findings obtained in this study delivered relevant information in the field of education and environmental psychology with respect to the relationships between parental socialization styles and empathy and connectedness with nature. These three variables were observed to be significantly related, thus enhancing knowledge in the field of education and environmental psychology, bearing in mind that little research has been conducted into these variables taken together. To some extent, these results served to confirm findings reported and highlighted in numerous scientific studies in the field of education and psychology—namely, the power that parental socialization styles have to enhance resources such as self-esteem, self-efficacy, and emotional intelligence. This study incorporated empathy and connectedness with nature. It was also observed that the indulgent and authoritative styles most enhanced empathy and environmental connectedness. These results are consistent with those reported in numerous studies in the field of psychology and education. Putting cultural differences aside, these results clearly confirm that indulgent and authoritative styles are the strongest enablers, and neglectful and authoritarian styles the weakest. Another noteworthy finding of this study was that empathy towards nature was not only stronger in women than men, but it was also independent of each parental socialization style, thus adding an interesting perspective to gender differences in environmentalism. However, men from authoritative and indulgent families showed greater empathy and connectedness with the environment, while those raised by neglectful and authoritarian parents were less empathetic, with significant differences with respect to women.

## Figures and Tables

**Table 1:** Numbers of cases in parenting style groups, as well as mean scores and standard deviations on measures of parental dimensions.

Variables	Total Sample *N* (%)	Parenting Style			
		Neglectful (*n* = 197) *N* (%)	Authoritarian (*n* = 166) *N* (%)	Indulgent (*n* = 227) *N* (%)	Authoritative (*n* = 207) *N* (%)
Sex					
Male	377 (47.3%)	91 (24.1%)	99 (26.3%)	97 (25.7%)	90 (23.9%)
Female	420 (52.7%)	106 (25.2%)	67 (16%)	130 (31%)	117 (27.9%)
Total	797 (100%)	197 (24.7%)	166 (20.8%)	227 28.5%)	207 (28.5%)
Acceptance/Involvement					
*Mean*	3.14	2.76	2.76	3.43	3.50
*SD*	0.45	0.31	0.29	0.26	0.24
Strictness/Imposition					
*Mean*	2.00	1.66	2.32	1.76	2.33
*SD*	0.39	0.22	0.27	0.23	0.26

**Table 2:** Multivariate analysis of variance (MANOVA) results for all the studied variables (4^a^ × 2^b^).

Variables		Source of Variation				
	*Λ*	*F*	*df_between_*	*df_error_*	*p*	*η* ^2^
(A) Parenting Style ^a^	0.919	7.514	9	1915.502	<0.001 ***	0.028
(B) Sex ^b^	0.948	14.290	3	787	<0.001 ***	0.052
A × B	0.987	1.921	9	1915.502	<0.05 *	0.004

a_1_, neglectful, a_2_, authoritarian, a_3_, indulgent, a_4_, authoritative; b_1_, male, b_2_, female. *** *p* < 0.001; * *p* < 0.05.

**Table 3:** Means (standard deviations) of parenting style, as well asmain univariate *F*values for connectedness to the natural environment, emotional empathy to natural environment, and cognitive empathy for natural environment.

Variables	Parenting Style					
	Neglectful	Authoritarian	Indulgent	Authoritative	*F*(3, 793)	*η* ^2^
CNE	3.18 (0.81) ^b^	3.22 (0.79) ^b^	3.55 (0.75) ^a^	3.48 (0.79) ^a^	11.526 ***	0.042
EENE	3.67 (0.89) ^b^	3.64 (0.84) ^b^	4.04 (0.65) ^a^	4.06 (0.79) ^a^	16.370 ***	0.058
CENE	3.25 (0.80) ^b^	3.29 (0.91) ^b^	3.66 (0.76) ^a^	3.61 (0.88) ^a^	12.994 ***	0.047

Note: CNE = Connectedness to the natural environment; EENE = Emotional empathy to natural environment; CENE = Cognitive empathy for natural environment. *** *p* < 0.001; a > b.

**Table 4:** Means (standard deviations), and main univariate *F*values for gender and connectedness to the natural environment, emotional empathy to natural environment, and cognitive empathy for natural environment.

Variables	Sex		*F*(1, 795)	*η* ^2^
	Male	Female		
CNE	3.35 (0.83)	3.39 (0.77)	0.416	0.001
EENE	3.67 (0.89)	4.05 (0.69)	46.401 ***	0.055
CENE	3.33 (0.91)	3.59 (0.78)	19.035 ***	0.023

Note: CNE = Connectedness to the natural environment; EENE = Emotional empathy to natural environment; CENE = Cognitive empathy for natural environment. *** *p* < 0.001; ** *p* < 0.01.

**Table 5:** Means (standard deviations), and main univariate *F*values for parenting style, gender and emotional empathy to natural environment.

Parenting Style							
Variables	Sex	Neglectful	Authoritarian	Indulgent	Authoritative	*F*(3, 789)	η^2^
EENE	**Male**	3.36 (0.97) ^c^	3.48 (0.84) ^c^	3.86 (0.74) ^b^	3.98 (0.88) ^a^	2.76 *	0.010
	**Female**	3.94 (0.72) ^a^	3.87 (0.79) ^a^	4.18 (0.54) ^a^	4.13 (0.71) ^a^		

Note: EENE = Emotional empathy to natural environment. * *p* < 0.05; a > b > c.
